# Impact of Psychosocial Stress in Early Life on Pace of Aging in Young Adulthood

**DOI:** 10.21203/rs.3.rs-6630823/v1

**Published:** 2025-05-23

**Authors:** Shaoyong Su, Tené T Lewis, Daniel W Belsky, Yutao Liu, Kai Zhang, Harold Snieder, Xiaoling Wang

## Abstract

Background: Early-life psychosocial stress is increasingly recognized as a contributor to accelerated biological aging and health disparities, yet its impact during young adulthood remains underexplored.​ Existing studies often focus on one or two dimensions of stress exposure and rely on retrospective assessments. Utilizing data from a longitudinal cohort initiated in 1989, we aim to examine the impact of early life psychosocial stress on accelerated aging in young adulthood, as well as its potential contribution to health disparities between Black and White Americans. Participants included 470 individuals (223 Black and 247 White Americans) with DNA samples collected at age > 20 years.​ Psychosocial stress exposures in the first 20 years of life were assessed prospectively using validated instruments across individual, family, and neighborhood domains. DunedinPACE, a novel biomarker of the pace of aging, was calculated from DNA methylation data generated from peripheral blood using the Illumina 450K array. The joint effect of early life psychosocial factors on DunedinPACE and the relative importance of each stressor were estimated using the Weighted Quantile Sum (WQS) approach. Mediation analysis was conducted to evaluate the contribution of early life stress to racial disparities in aging. Results: Compared to White Americans, Black Americans reported higher overall levels of early life stress (mean = 1.54 vs. 1.40, adjusted p = 0.003) and exhibited a faster pace of aging in young adulthood (DunedinPACE z-score mean = 0.21 vs. -0.19, adjusted p < 0.001). WQS analysis revealed a positive joint effect of early life psychosocial stress on DunedinPACE (β = 0.26, 95% CI: 0.167–0.353), with the top four contributors being parental socioeconomic status, peer pressure, family emotional expression, and neighborhood safety conditions. Mediation analysis indicated that early-life stress accounted for 22% of the racial disparity in biological aging (p = .01).​ Conclusion: Our findings suggest that exposure to disadvantaged psychosocial environments in early life is associated with accelerated biological aging in young adulthood. Racial differences in stress exposure partially explain disparities in aging, underscoring the importance of early interventions to reduce health disparities across the life course.

### BACKGROUND

Increasing evidence highlights the profound influence of chronic psychosocial stress on health and overall well-being. [1] This is particularly critical during childhood and adolescence, a dynamic period characterized by rapid physical, cognitive, and emotional development. [2] Adverse conditions, such as poverty, family conflict, unsafe neighborhoods, and discrimination, can create stressful living environments that disrupt developmental processes and lead to long-term consequences. [3, 4] These early-life experiences not only affect immediate well-being but also shape health trajectories over the lifespan, contributing to persistent disparities in physical and mental health. [5] Despite growing recognition of these impacts, much of the existing research has been limited to one or two dimensions of stress assessment, often relying on retrospective or cross-sectional data. This underscores the urgent need for comprehensive multidimensional approaches and longitudinal studies to better understand the complexity of early life stress (ELS), its cumulative effects on health, and the underlying mechanisms.

One plausible mechanism linking ELS to adverse health outcomes is accelerated biological aging. [6, 7] Although there is no gold-standard measurement of biological aging, [8] integration of omics data and machine-learning methods have yielded a new family of algorithm-based biomarkers that show substantial promise. [9, 10] Among these, algorithms derived from analysis of DNA methylation, commonly referred to as “epigenetic clocks”, are among the most widely studied. [11] Of these, several have accumulated substantial validation evidence as reliable predictors of healthspan and lifespan. [12] In addition, the best-validated clocks also exhibit reliable correlations with a range of social determinants of health, including stress exposure. [6] In particular, the DunedinPACE clock stands out as the clock most consistently associated with stress exposures in young people. [13–17] However, prospective data collected from childhood remain limited. [13, 14, 18] Furthermore, health disparities in age-related diseases and racial differences in ELS are large and persistent with Black Americans carrying a larger burden than White Americans in the United States. [19–22] Yet, how ELS contributes to Black-White differences in health outcomes during young adulthood using accelerated biological aging as an index remains poorly understood.

To address these gaps, we utilized data from a well-established longitudinal cohort featuring assessments across the lifespan: the Georgia Stress and Heart (GSH) study. [23] This cohort includes approximately equal numbers of Black and White Americans, as well as males and females. It has prospectively collected measures of psychosocial stress from a multidimensional perspective using a wide range of instruments covering individual, family and neighborhood psychosocial exposures. Notably, these factors tend to co-occur in accordance with socioeconomic patterning and often correlate with each other. Thus, we adopted a novel approach recently developed for studying the health effects of chemical mixtures, in which not only the tendency for clustering or co-occurrence of multiple factors is acknowledged but also the joint effect and the relative importance of multiple exposures are evaluated. [24] Leveraging the GSH cohort, the current study aims to examine the associations between ELS, DNA methylation-based pace of aging, and racial disparities, integrating comprehensive stress assessments with a prospective design.

## METHODS

### Subjects

All participants were from the Georgia Stress and Heart (GSH) study, an ongoing longitudinal cohort that was established in 1989 to study the development of cardiovascular risk factors from childhood to adulthood. Cohort design and selection criteria have been described previously. [23] Briefly, participants who met the following criteria were recruited: (1) aged 5 to 16 years in 1989; (2) Black American or White American; (3) normotensive for age and sex based on BP screening; and (4) free of chronic disease based on parental reports of the child's medical history. Participants were classified as Black Americans if both parents reported being of African heritage and they considered themselves and their child to be African American, black, or Afro-American. Participants were classified as White Americans if both parents reported that they were of European ancestry and they considered themselves and their child to be European American, white, or Caucasian, and not of Hispanic, Native American, or Asian descent. Evaluations were conducted annually from 1989 to 2000 (visit 1-10), every 1.5 year from 2002 to 2006 (visit 11-14) and every 2 years from 2008 to 2012 (visit 15-16).

In the present study, we selected subjects who had DNA samples available at their most recent visit when they were >20 years old. This is defined as the outcome visit at which genome-wide DNA methylation data was generated. A total of 470 subjects were identified. The Institutional Review Board at the Medical College of Georgia had given approval for the study. Informed consent was provided by all subjects, or by parents if subjects were <18 years old.

#### Assessments of Psychosocial Stress

A battery of validated measures of exposure to chronic psychosocial stress, such as stressful life events, perceived discrimination, family environment, and neighborhood safety, were collected through questionnaires since visit 7. Details are shown in Supplemental Table 1.

The Adolescent Resources Challenges Scale (ARCS) [25] is a self-report scale that assesses whether the respondent has experienced any stressful events during the past 12 months, including stressful life experiences from the *neighborhood environment* (e.g., "I saw or heard people my age or older fighting on the street where I live" and "I saw or heard someone dealing drugs in my neighborhood"), *family* (e.g., family members abused alcohol or drugs), and *peer environment* (e.g., "I argued or had problems with my boyfriend/girlfriend" and "my friends got drunk or used drugs"). Three subscales were scored to indicate sources of stress from peers (ARCS-Peer), family (ARCS-Family) and neighborhood (ARCS-Neighbor).

The Day-to-Day Perceived Discrimination Scale (PDS) [26] and the Unfairness Scale (UNF) [27] were used to assess chronic, routine experiences of discrimination and unfair treatment due to factors such as ethnicity, social class, physical appearance, or sex in daily life.

The Family Environment Scale (FES) [28] was used to assess familial stress and functioning in which the respondent was living. Three subscales were calculated. *Cohesion (FES-Coh)* refers to the degree of commitment, help and support family members provide for one another. *Expressiveness (FES-Exp)* refers to the extent to which family members are encouraged to act openly and to express their feelings directly. *Conflict (FES-Cnf)* refers to the amount of openly expressed anger, aggression and conflict among family members.

The Hollingshead Four-Factor Index of Social Status (Hollingshead) [29] was used as the index of family socioeconomic status (SES). Briefly, a score was calculated based on the educational attainment and occupational level of the parents. Both father's and mother's SES were calculated, and the higher value was used to represent family SES. This information has been collected since the baseline, and the average value calculated across all visits was used to represent the overall childhood SES.

The Adverse Childhood Experiences (ACE) [30] questionnaire consists of 28 items divided into 3 categories, including childhood abuse (emotional, physical and sexual), neglect (emotional and physical), and growing up with household dysfunction (substance abuse, mental illness, domestic violence, criminal household member, and parental marital discord). This questionnaire was administered only at visit 15 at which all participants were older than 19 years. A total score (ranged 0-10) was calculated to assess traumatic experiences prior to age 18.

Aggregation of exposure to chronic psychosocial stress prior to age 20. Except for the Hollingshead Index (available for all visits) and the ACE (available for a single visit), the other questionnaires were administered multiple times during different visits between visit 7 and visit 16 (Suppl Table 1). We included all six instruments to measure participant's psychosocial stress in the first 20 years of life, which covers key transition periods from childhood to adolescence to young adulthood during which a person is still undergoing physiobiological and psychological development. Based on these six instruments, 10 subscales were generated to comprehensively assess the participant's exposures to psychological and social environments in four domains: childhood SES, individual stressful events, family environment, and neighborhood safety (Suppl Table 2). To maximize the use of psychosocial stress data assessed on multiple levels (i.e., individual, family, and neighborhood) across all visits, we included all participants who had at least one assessment for any of the 10 subscales before age 20. For each participant, if one subscale (except for Hollingshead and ACE) was administered in more than one visit, a z-score was calculated for each visit and the average score across the visits was calculated to represent the overall exposure of that subscale before age 20. Out of the 470 participants who had DNA methylation data available, 97% have at least 6 of 10 subscales and 77% have non-missing on all 10 subscales. For each subscale, the missing value was between 0%-9% for the 470 participants, except for ACE, which was available in 85% of participants. We applied a multiple imputation approach [31] to impute all missing values for each subscale. The imputed values were used in the subsequent data analysis.

#### Biological Aging

We measured the pace of biological aging using DunedinPACE, a DNA methylation algorithm. [18] DunedinPACE was developed within the Dunedin Longitudinal Study from analysis of two-decades of longitudinal data on 19 biomarkers of the functioning of a range of organ systems. Unlike other

“epigenetic clocks”, which aim to summarize the lifetime extent of biological aging, DunedinPACE measures the current rate at which biological aging is occurring. DunedinPACE has an expected value of 1 in midlife adults, corresponding to the average years of biological deteriation experienced during a 12-month calendar interval. DunedinPACE values can be interpreted as the ratio of years of biological aging per 12 months of calendar time (e.g. a value of 1.2 corresponds to a 20% faster pace of aging vs. the norm). In this way, DunedinPACE functions like a speedometer for the aging process. We focused on the DunedinPACE measure of biological aging because of our expectation that, among young adults, it would be the pace of aging that would be most impacted by life stress.

We calculated DunedinPACE using the R package accessed via GitHub *(https://github.com/danbelsky/DunedinPACE)* from peripheral blood DNA methylation data generated with the Illumina Infinium Human Methylation 450K Beadchip (Illumina Inc.). For analysis, DunedinPACE values were scaled to have M = 0 and SD = 1. A detailed description of preprocessing and quality control steps has been described previously. [32]

#### Covariates

At each visit, subject’s height and weight were measured with a Healthometer medical scale that was calibrated daily. Body mass index (BMI, $kg/m^2$) was calculated as a measure of general adiposity. Participant’s smoking status was assessed by self-report. Subjects who smoked >1 cigarette in the past 30 days were considered as smokers. In addition to race and sex, age, BMI, and smoking status at the visit when DNA was collected were included as covariates in the subsequent data analysis. Cell type compositions (B lymphocytes, CD4+ T cells, CD8+ T cells, monocytes, natural killer cells, and neutrophils) were estimated using the approach described previously. [32] We also adjusted for the proportions of cell types to reduce the potential confounding from cell compositions.

#### Statistical Analysis

All descriptive analyses were performed using STATA software. Racial differences in general characteristics were examined (adjusted for age and sex) by using linear regression for continuous traits, logistic regression for binary traits, or ordinal logistic regression for categorical traits, repectively. For each stressor, the values were categorized into quantiles. Of note, protective factors such as high family SES (indexed by parental Hollingshead score) and friendly family environment (indexed by FES-Cohesion and FES-Expression) were reverse coded, so that the higher score represents the worse situation. Spearman correlations were calculated among these factors.

The Weighted Quantile Sum (WQS) approach [24] was used to examine the joint effect of psychosocial stress in early life on epigenetic pace of aging (DunedinPACE z-score) and to estimate the relative importance of each stress exposure. Briefly, WQS is a statistical model for multivariate regression in a high-dimensional dataset that operates in a supervised learning framework. The WQS score is calculated as a weighted sum (so that exposures with weaker effects on the outcome have lower weight in the index) of all exposures categorized into quartiles, so that extreme values have less impact on the weight estimation. A WQS regression model takes the following form: $g(\mu)=\beta_0+\beta_1(\ )+z'\varphi$. The   term represents the index that sums the weighted (w) exposures (q) (the number of exposures is k) included in the mixture; $\beta_1$ is the parameter summarizing the joint effect of the mixture; $z'$ is the vector of covariates, $\varphi$ is the vector of parameters associated with the covariates, and $g(\mu)$ is the link function. The individual weight $w_i$ indicates the relative importance of the $i^{th}$ exposure in the mixture-outcome association. A cut-off value was established to identify the most important elements of the mixture and set equal to the inverse of the number of the mixture components (1/10 = 0.10). A repeated holdout validation combining cross-validation and bootstrap resampling approach was conducted to produce stable and optimistic results. The model is one-directional, in that it tests for mixture effect positively associated with epigenetic aging. The WQS model was performed with the following parameters: (1) age, sex, race, BMI, and smoking as covariates; (2) a 40-60 training validation split; (3) positive direction; and (4) a repeated holdout with 100 repeated holdout validation and 100 bootstrap samplings. [33] The R package gWQS was used for the WQS regression analysis.

We further performed mediation analysis to test whether $WQS_{ELS}$ can mediate the racial difference in DunedinPACE. [34] We used bootstrapping to test for mediation significance. The covariates were included in all models. Mediation was considered to be present when racial difference in pace of aging decreased upon the addition of the early life stress WQS score to the model and the bootstrap test gave $p \leq 0.05$.

## RESULTS

Descriptive characteristics of subjects at their most recent outcome visit are presented in Table 1 by race. Compared to White American participants, Black American participants were slightly older (average age of 28 vs. 27, p<0.001), and had a higher BMI (31.5 vs. 28.7, p=0.008), as well as a lower rate of ever smoking cigarettes (25% vs. 35%, p=0.06).

As shown in Table 1, Black American participants reported a higher rate of low parental SES levels than White American participants (37% vs. 14% in the $4^{th}$ quantile, p<0.001). Significant racial differences are evident in some of the exposures to psychosocial stress in early life. For instance, Black Americans experienced more exposures to unfair treatment (30% vs. 20%, p=0.001) and ACEs (23% vs. 17%, p=0.02) than White Americans, while White Americans reported a higher rate of experiencing peer pressure (34% vs. 15%, p<0.001) and overall familial stress (29% vs. 20%, p=0.04). However, there were no substantial differences in rates of strained family relationships between White and Black American participants. Concerning neighborhood safety, Black American participants experienced a higher rate of unsafe conditions in their neighborhoods compared to their White American counterparts (31% vs. 19%, p<0.001).

As expected, there are substantial correlations among psychosocial stress factors (Figure 1). For example, a strong correlation was found between ARCS_Family and ARCS_Peer (r=0.49, p<0.001), FES_Coh and FES_Cnf (0.66, p<0.001), and PDS and UNF (r=0.52, p<0.001), respectively. ACE exposures were highly correlated with family stress and functioning indexed by FES_Coh (r=0.46, p<0.001) and FES_Cnf (r=0.38, p<0.001). Neighborhood safety conditions (ARCS_Neighbor) were moderately correlated with other exposures (r=0.18-0.40, p<0.001). Interestingly, parental SES (Hollingshead) was correlated with some of the stressors, but not all.

A total of 100 repeated holdout WQS regressions were performed, along with 100 bootstrap samplings. Table 2 presents the effect of the WQS index on the pace of aging, along with the 95% Confidence Intervals (CIs). After adjusting for covariates, a significant joint effect was observed (β=0.26, 95% CI: 0.167 – 0.353), indicating that a distressed living environment in early life may accelerate biological aging in young adulthood. Given the distribution of the WQS index (M = 1.47, SD = 0.60), a one SD increase in WQS is associated with a 0.16 SD increase in DunedinPACE. Table 2 also shows the average weights of each psychosocial factor contributing to the WQS index, where parental SES (Hollingshead), peer pressure (ARCS_Peer), family environments (FES_Exp), and neighborhood safety conditions (ARCS_Neighbor) had greater weights than the prespecified cutoff (1/10 = 0.10). Supplementary Figure 1 illustrates the box-plot of the weights for each exposure contributing to the WQS index estimated through the 100 repeated holdout WQS regressions. Further adjustment for cell compositions did not change the results.

The WQS index for early life stress ($WQS_{ELS}$) was calculated based on the average weights of each stressor, with the distribution by racial group shown in Figure 2. The overall distribution for Black Americans is shifted to the right compared to White Americans, with mean values (SD) of 1.54 (0.60) and 1.40 (0.60), respectively (adjusted p=0.003). This shift suggests that Black Americans experienced higher exposure to early life stress than White Americans.

There was also a significant difference in the DNA methylation pace of aging between Black American and White American participants (Table 1), with Black Americans showing a higher DunedinPACE value than White Americans (mean z-score = 0.21 vs. -0.19, age- and sex-adjusted β = 0.32, adjusted p<0.001).

Mediation analysis indicated that adding $WQS_{ELS}$ to the model attenuated the association between race and DNA methylation pace of aging (Figure 3). A bootstrapping test confirmed that the mediation effect was significant (p=0.01), with $WQS_{ELS}$ explaining 22% of the racial difference in epigenetic aging.

## DISCUSSION

This study appears to be the first to examine the association between early-life psychosocial stress and accelerated aging in young adulthood using aggregated longitudinal stress measurements. We found that a distressed living environment in early life was associated with accelerated biological aging in young adulthood. Black participants exhibited more advanced biological aging and carried a greater ELS burden compared to their White counterparts. In mediation analysis, the higher ELS burden among Black Americans accounted for up to 22% of the racial difference in biological aging. Our findings align with previous research demonstrating that ELS can accelerate biological aging, offering further validation from a longitudinal life-course study that prospectively and comprehensively captured ELS. This study also provides proof of the concept that racial disparities in early-life social and environmental stressors contribute to health disparities in young adulthood measured by pace of methylation aging.

The evidence consistently supports the association between ELS and accelerated biological aging, with low childhood SES and adversity being the most frequently studied exposures. Most research to date has relied on adult DNA methylation data coupled with retrospective measures of ELS. [17, 35–38] For example, in the Health and Retirement study, participants who reported higher parental education exhibited slower epigenetic aging. [39] Similarly, a meta-analysis of adult populations found that retrospectively reported childhood trauma exposure was associated with advanced epigenetic age. [40]

The relationship of childhood SES and adversity with biological aging has also been explored in cross-sectional studies of children, yielding similar findings. [14, 41] However, studies that prospectively recorded ELS during childhood and assessed epigenetic aging in young adulthood remain limited. In the E-Risk Longitudinal Study, children raised in lower SES families or exposed to multiple types of victimization exhibited a faster pace of aging at age 18. [18] Similarly, in the ALSPAC birth cohort, childhood adversity measured from birth to age 14 was linked to advanced epigenetic age at age 17. [42] The Great Smoky Mountains Study further demonstrated that childhood adversity was associated with accelerated within-person changes in methylation age from adolescence to young adulthood. [43] Additionally, the Strong African American Families Healthy Adults Project found that children raised in emotionally unsupportive and harsh family environments (i.e., risky family climates) exhibited advanced epigenetic aging in young adulthood. [44]

A unique aspect of our study is its prospectively collected, multidimensional assessment of childhood psychsocial stress exposures, incorporating a broad range of measures at the individual, family and neighborhood levels. It has long been realized that pyschosocial stress is a complex phenomenon encompassing various interrelated components, including low SES, disadvantaged living conditions, trauma experiences, stressful life events, family dysfunction, perceived unfair treatment, and discrimination - factors that often co-occur along socioeconomic patterning. However, prior research has not comprehensively examined the cumulative impact of these exposures on biological aging in adulthood or their relative contributions. By employing a novel approach recently developed for studying the health effects of chemical mixtures, our study provides new insights into the effects of early life psychosocial stress. We not only observed that a distressed early life living environment increased the risk of advanced biological aging in young adulthood but also identified key contributing factors, including parental SES, peer pressure, family emotional expression, and neighborhood safety conditions.

Another unique aspect of our study was its biracial design, recruiting both Black and White Americans. Health disparities are well-documented in epidemiologic statistics, with Black Americans experiencing higher rates of multiple age-related disorders and lower life expectancy than White Americans. Studies using second- or third-generation epigenetic aging markers have consistently shown that Black

Americans exhibit faster or more advanced biological aging than White Americans. [45] However, the underlying mechanisms driving these disparities remain unclear. Social and economic disadvantages, along with the lifelong stress of coping with adversity, have been proposed as key contributors. [46] Addressing this question requires a multi-level, life-course approach, as disparities likely arise from the accumulation of small differences over time. [47] For the first time, our study provides supporting evidence for this hypothesis, demonstrating that Black participants not only experienced a higher burden of ELS but that this burden accounted for up to 22% of Black-White differences in biological aging in young adulthood.

This study has several limitations. First, missing data on psychosocial stress assessments were addressed using multiple imputation, with minimal missingness (0–9%) except for ACE. A sensitivity analysis restricted to participants with available ACE data yielded consistent results, suggesting imputation did not bias findings. Second, our mixture analysis assumed that greater ELS exposure accelerates biological aging, an assumption strongly supported by existing literature. We then conducted a sensitive test by allowing both positive and negative indices in the model and observed a statistically significant association in the positive but not in the negative direction, which confirmed our assumption. Third, in this study, we relied on individual perceptions of neighborhood social and environmental factors rather than objective exposure measurements, as individual-level measures typically show stronger associations with health outcomes. However, recent advances in precision public health now enable the capture of living conditions at more granular geographic levels (e.g., neighborhood conditions), enhancing our ability to understand how contextual and personal factors interact to influence health. Future studies will incorporate these detailed geographical measures to further investigate the impact of neighborhood disadvantages. Lastly, in the current study, biological age was estimated through one-time measurement of DNA methylation in young adulthood. Although the approach we used, i.e., DunedinPACE, gave an estimation of pace of aging rather than biological age relative to chronological age, future analysis incorporating multiple time-point DNA methylation data obtained through follow up of the cohort into middle age will provide more evidence on the long-term impact of ELS on accelerated aging and deepen our understanding of how ELS influences health across the life course.

## CONCLUSIONS

In summary, leveraging a unique longitudinal cohort with multiple domains of early life childhood psychosocial stress prospectively collected in both Black and White Americans, along with a novel statistical approach, our study expands previous research on ELS and biological aging by demonstrating the cumulative overall effect of ELS and its mediating role on health disparities in later life.

## Abbreviations

ACE, Adverse Childhood Experiences; ARCS, Adolescent Resources Challenges Scale; BMI, body mass index; ELS, early life stress; FES, Family Environment Scale; FES-Coh, FES-Cohesion; FES-Cnf, FES-Conflict; FES-Exp, FES-Expressiveness; GSH, Georgia Stress and Heart; HOLLINGSHEAD, Hollingshead

Four Factor Index; PDS, Perceived Discrimination Scale; SES, socioeconomic status; UNF, Unfairness Scale; WQS, weighted quantile sum

## Declarations

#### *Ethics approval and consent to participate*

The Institutional Review Board at the Medical College of Georgia had given approval for the study. Informed consent was provided by all subjects, or by parents if subjects were <18 years old.

#### *Availability of data and materials*

All data used for this study will be available upon request.

#### *Conflic of Interest*

Dan Belsky is an inventor of DunedinPACE, which is licensed by Duke University and the University of Otago to TruDiagnostic, from which he receives royalties. DunedinPACE is freely available for scientific research.

#### *Funding*

This study was supported by Grants HL069999 and HL143440 from the National Institute of Health.

Harold Snieder acknowledges ‘Stress in Action’ (www.stress-in-action.nl) funded by the Dutch Research Council and the Dutch Ministry of Education, Culture and Science (NWO gravitation grant number 024.005.010).

Dan Belsky is a fellow of the Canadian Institute for Advanced Research CBD Network.

#### *Authors' contributions*

Shaoyong Su: Conceptualization, Formal analysis, Writing - Original Draft; Tené T Lewis: Writing - Review & Editing; Daniel W Belsky: Writing - Review & Editing; Yutao Liu: Writing - Review & Editing; Kai Zhang: Writing - Review & Editing; Harold Snieder: Writing - Review & Editing; Xiaoling Wang: Writing - Review & Editing

#### *Acknowledgements*

We would like to thank all the participants in the study and the staff at the Georgia Prevention Institute.

## Tables

**Table 1.** General characteristics, exposure, and outcome data of the subjects at their most recent visit (N=470)

| | White American (N=247) | Black American (N=223) | P values* |
|---|---|---|---|
| **Demographic** | | | |
| Age, mean (SD) | 27.0 (3.5) | 28.0 (3.1) | **0.001** |
| Female, n (%) | 118 (48%) | 127 (57%) | 0.07 |
| BMI (kg/m2), mean (SD) | 28.7 (7.7) | 31.5 (8.6) | **0.008** |
| Ever smoking (yes), n (%) | 86 (35%) | 55 (25%) | 0.06 |
| **Cumulative Exposure to Early-life Stress** | | | |
| ***Family SES*** | | | |
| Parental Hollingshead score >75$^{th}$, n (%) † | 34 (14%) | 82 (37%) | **<0.001** |
| ***Stressful Life Events*** | | | |
| ARCS-Peer >75$^{th}$, n (%) | 83 (34%) | 34 (15%) | **<0.001** |
| ACE >75$^{th}$, n (%) | 41 (17%) | 52 (23%) | **0.02** |
| PDS >75$^{th}$, n (%) | 57 (23%) | 60 (27%) | 0.33 |
| UNF >75$^{th}$, n (%) | 49 (20%) | 68 (30%) | **0.001** |
| ***Family Environment*** | | | |
| ARCS-Family >75$^{th}$, n (%) | 72 (29%) | 45 (20%) | **0.04** |
| FES-Coh >75$^{th}$, n (%) † | 62 (25%) | 55 (25%) | 0.16 |
| FES-Exp >75$^{th}$, n (%) † | 61 (25%) | 56 (25%) | 0.07 |
| FES-Cnf >75$^{th}$, n (%) | 64 (26%) | 53 (24%) | 0.56 |
| ***Neighborhood Environment*** | | | |
| ARCS-Neighborhood >75$^{th}$, n (%) | 47 (19%) | 69 (31%) | **<0.001** |
| **Epigenetic Aging Marker** | | | |
| DunedinPACE (z score), mean (SD) | -0.19 (0.99) | 0.21 (0.96) | **<0.001** |

* P values were adjusted for age and sex. Linear, logistic and ordinal logistic regressions were used for continuous, binary, and categorical variables respectively. P<0.05 were in bold.

† The score was reverse coded.

**Table 2.** WQS regression results: the estimate and 95% confidence intervals (CI) of the WQS index on DunedinPACE (z-score) are shown. The second part of the table shows the average magnitude of the weights and 95% CIs for each stressor contributing to the WQS index.

| WQS Index | Estimate | 95% CI | |
|---|---|---|---|
| $WQS_{ELS}$ | 0.26 | 0.167 | 0.353 |

| Mixture Component | Weights | | |
|---|---|---|---|
| Parental_Hollingshead | 0.204 | 0.063 | 0.374 |
| ARCS_Peer | 0.138 | 0.009 | 0.366 |
| FES_Exp | 0.134 | 0.014 | 0.303 |
| ARCS_Neighbor | 0.131 | 0.012 | 0.286 |
| FES_Cnf | 0.095 | 0.005 | 0.242 |
| UNF | 0.092 | 0.003 | 0.213 |
| PDS | 0.064 | 0.002 | 0.204 |
| ARCS_Family | 0.061 | 0.003 | 0.193 |
| FES_Coh | 0.044 | 0.000 | 0.170 |
| ACE | 0.036 | 0.000 | 0.159 |

## Figures

| | | | | | | | | | | |
|---|---|---|---|---|---|---|---|---|---|---|
| **ARCS_Family** | 1.00 | | | | | | | | | |
| **ARCS_Peer** | 0.49 | 1.00 | | | | | | | | |
| **ARCS_Neighbor** | 0.36 | 0.34 | 1.00 | | | | | | | |
| **FES_Coh** | 0.30 | 0.17 | 0.31 | 1.00 | | | | | | |
| **FES_Exp** | 0.18 | 0.08 | 0.18 | 0.42 | 1.00 | | | | | |
| **FES_Cnf** | 0.27 | 0.18 | 0.29 | 0.66 | 0.32 | 1.00 | | | | |
| **PDS** | 0.40 | 0.27 | 0.35 | 0.27 | 0.27 | 0.33 | 1.00 | | | |
| **UNF** | 0.29 | 0.25 | 0.40 | 0.27 | 0.20 | 0.27 | 0.52 | 1.00 | | |
| **ACE** | 0.18 | 0.13 | 0.31 | 0.46 | 0.24 | 0.38 | 0.26 | 0.27 | 1.00 | |
| **Parental_Hollingshead** | -0.03 | -0.07 | 0.24 | 0.27 | 0.14 | 0.19 | 0.00 | 0.06 | 0.36 | 1.00 |

**Figure 1**

Spearman correlations among the psychosocial stress factors.

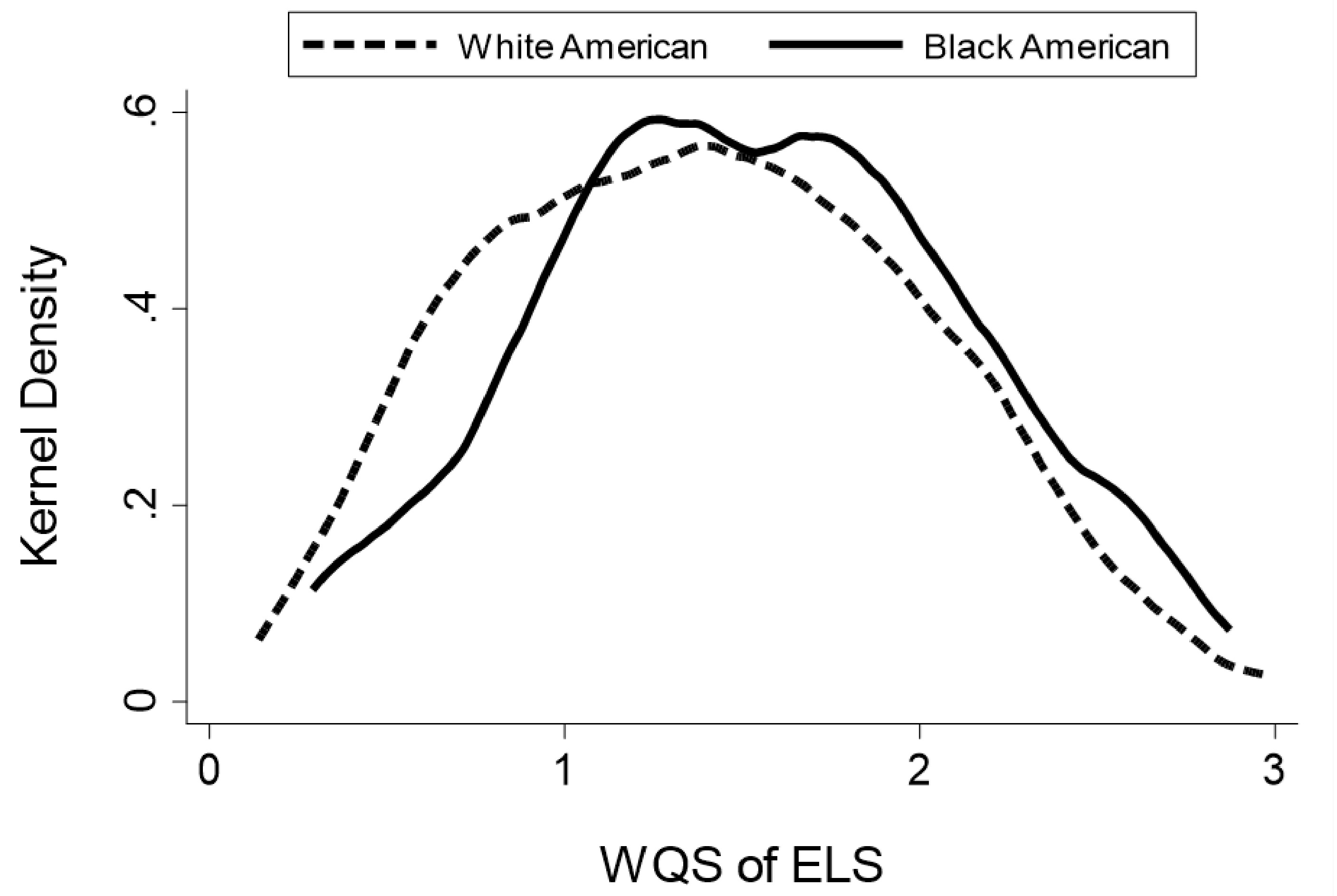

**Figure 2**

Distribution of WQS index of early life stress (ELS) by White Americans and Black Americans.

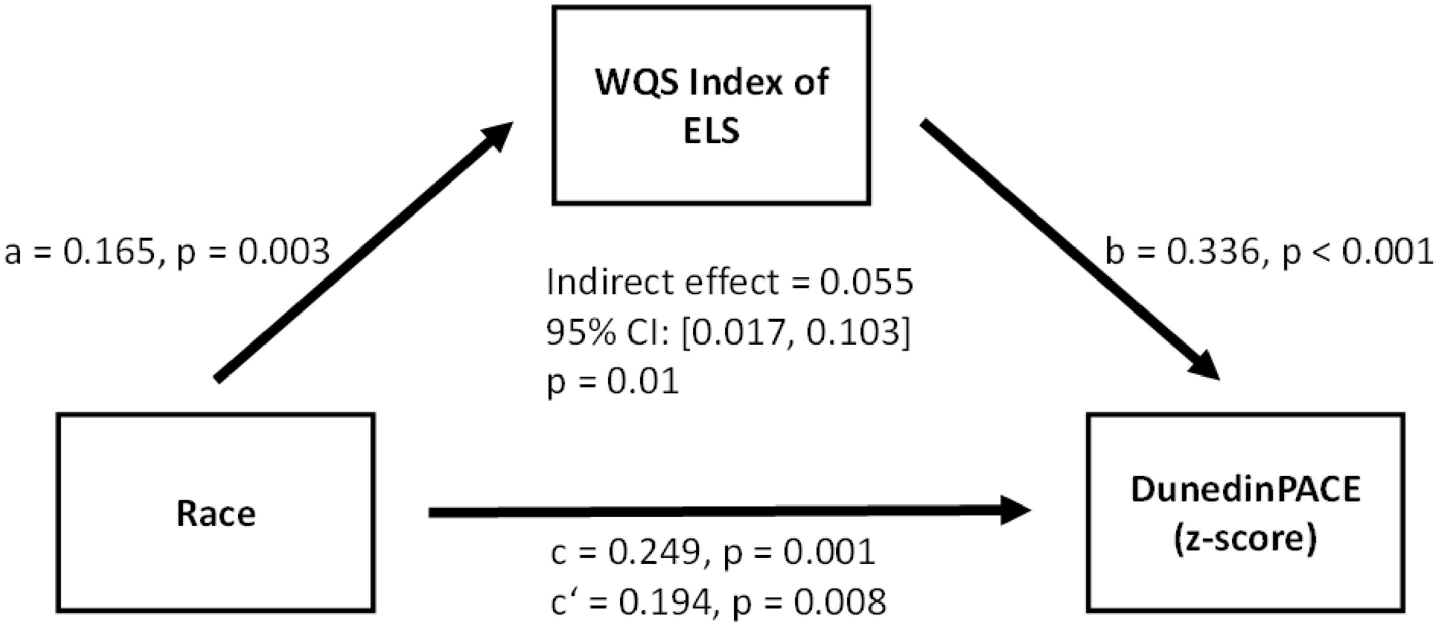

**Figure 3**

Partial mediation model illustrates early life stress ($WQS_{ELS}$) mediating the racial difference in DNA methylation pace of aging. Regression coefficients and adjusted p values are presented. Indirect effect was tested using 500 bootstrap samplings.

## Supplementary Files

This is a list of supplementary files associated with this preprint. Click to download.

- Supplemental.docx
